# Gene Expression Analysis of Pak Choi in Response to Vernalization

**DOI:** 10.1371/journal.pone.0141446

**Published:** 2015-10-30

**Authors:** Mengxia Sun, Xianhui Qi, Leiping Hou, Xiaoyong Xu, Zhujun Zhu, Meilan Li

**Affiliations:** 1 College of Horticulture, Shanxi Agricultural University, Taigu, Shanxi, China; 2 Key Laboratory for Quality Improvement of Agricultural Products of Zhejiang Province/College of Agricultural and Food Science, Zhejiang A&F University, Hangzhou, 311300, P. R. China; Institute of Crop Sciences, CHINA

## Abstract

Pak choi is a seed vernalization-type plant whose vernalization mechanism is currently unclear. Therefore, it is critical to discover genes related to vernalization and research its functions during vernalization in pak choi. Here, the gene expression profiles in the shoot apex were analyzed after low temperature treatment using high-throughput RNA sequencing technology. The results showed that there are 1,664 and 1,192 differentially expressed genes (DEGs) in pak choi in cold treatment ending and before flower bud differentiation, respectively, including 42 genes that exhibited similar expression trend at both stages. Detailed annotation revealed that the proteins encoded by the DEGs are located in the extracellular region, cell junction and extracellular matrix. These proteins exhibit activity such as antioxidant activity and binding protein/transcription factor activity, and they are involved in signal transduction and the immune system/biological processes. Among the DEGs, Bra014527 was up-regulated in low temperature treatment ending, Bra024097 was up-regulated before flower bud differentiation and Bra035940 was down-regulated at both stages in low temperature-treated shoot apices. Homologues of these genes in *A*. *thaliana*, AT3G59790, AT4G30200 and AT5G61150, are involved in flowering and vernalization, suggesting that they take part in the vernalization process in pak choi. Further pathway enrichment analysis revealed that most genes were enriched in the tryptophan metabolism and glucosinolate biosynthesis pathways. However, the functions of tryptophan and glucosinolate in vernalization are not yet clear and require further analysis.

## Introduction

Pak choi (*Brassica rapa* ssp. *chinensis* Makino) is a nutrient-rich cruciferous (*Brassica*) crop [1] that plays an important role in the annual vegetable supply. Pak choi is a seed vernalization-type plant that likely undergoes early bolting during production due to low temperatures in the Spring, leading to decline in commercial quality and causing economic losses. Therefore, it is crucial to study the vernalization mechanism in pak choi.

To date, the vernalization mechanism of the model plant *Arabidopsis thaliana* has been studied extensively. In *Arabidopsis thaliana*, *FLC* and *FRI* are flowering suppressor genes that play a vital role in the flowering process [2, 3], while *VIN3*, *VRN5*, *VRN1* and *VRN2* are regulatory genes that are directly related to vernalization [4]. VIN3 induces deacetylation of *FLC* histone H3 and reduces the expression of *FLC* at the beginning of vernalization [5], while *VRN1* and *VRN2* maintain low level expression of *FLC* during the late period of vernalization [6, 7]. Some studies of the vernalization mechanism in wheat have also been performed. Vernalization in wheat is mainly controlled by *VRN1*, *VRN2* and *VRN3*; *VRN1* is the main determining factor for the diversity of wheat vernalization growth characteristics [8], *VRN2* is a floral repressor gene [9] and *VRN3* is induced under long-day conditions to improve the reproductive development of wheat [10]. Some genes related to vernalization such as *VRN1 and VRN2* in wheat have been cloned and their expression characteristics have been investigated [11]. Yin et al. [12] found that soluble protein levels increase and some new proteins are generated during the vernalization process in wheat. In addition, the vernalization mechanisms in radish [13] and broccoli [14] have been investigated, but similar in-depth studies have not been performed in pak choi, and new genes related to vernalization remain to be discovered.

RNA-Sequencing (RNA-Seq) is a technology that combines high throughput sequencing with computer analysis. This technique is a highly accurate, high throughput technique with a wide detection range and excellent repeatability [15–17]. It has been used to analyze gene expression patterns and metabolic changes under heat stress [18], osmotic stress [19] in corn, which lays the foundation for discovering stress-related genes that function under poor conditions. Wang et al. [20] analyzed specifically expressed genes in soybean roots and leaves under salt stress using RNA-Seq and found that the ABC transporter and glutathione biosynthesis pathways may act synergistically under stress conditions. Chen et al. [21] analyzed soybean seeds at different developmental stages using RNA-Seq and discovered 12 genes involved in the fatty acid biosynthesis pathway. In addition, RNA-Seq has been used to study physiological metabolism under specific conditions in grape [22], cassava [23], cotton [24] and shrimp [25], but similar studies in pak choi have not been reported.

Full genome sequencing of Chinese cabbage has recently been completed[26]. In this study, using the Chinese cabbage genome as a reference, the gene expression profiles were analyzed in pak choi during the vernalization process using RNA-Seq technology to discover genes and metabolic pathways related to vernalization. The results of this study will facilitate the research on vernalization mechanism of the plant in the future.

## Materials and Methods

### Plant materials and RNA isolation

The pak choi inbred line ‘75^#^’ was used as the material in this study because it bolts easily. Seeds for this line were obtained from the Institute of Vegetable Research, Shanxi Academy of Agricultural Sciences. The experiment sets two treatments: low temperature and control treatment. For low temperature treatment, the germinating seeds were put at 4°C for 20 d, while the seeds under control treatment germinated and grew normally. The seedlings were transplanted to the substrate at the same time when they were of the same size and at the same growth stage. Shoot apices with cotyledons from both types of plants were sampled separately before transplanting. Each 0.15 g sample was collected from six seedlings and the samples for low temperature and control treatment were designated V and CK respectively. The plants were cultivated in conventional way, and flower bud differentiation was observed during growth and development. Flower buds of the vernalized plants began to differentiate at 16 d after transplanting, which did not occur in the control plants. At 15 d after transplanting, immediately prior to differentiation of the vernalized flower buds, the shoot apices of both plants were sampled as above, producing samples V15 and CK15. All samples were frozen in liquid nitrogen and stored at -80°C until use.

Total RNA was extracted from each sample using an RNeasy Plant Mini Kit (QIAGEN, 74903) following the manufacture’s instructions. The quality and quantity of the RNA were determined by gel electrophoresis and the use of a NanoDrop 1000 spectrophotometer. The RNA was stored at -80°C for subsequent analysis.

### cDNA library construction and sequencing

The mRNA from the total RNA samples was enriched using oligo magnetic adsorption, and the resulting RNA was fragmented. The RNA fragments served as a template for first-strand cDNA synthesis using random hexamers and reverse transcriptase. Second-strand cDNA was synthesized using DNA polymerase I and RNaseH and purified using a QiaQuick PCR extraction kit. Finally, cDNA fragments of a suitable length (300–500 bp) were obtained by agarose gel electrophoresis and amplified by PCR to construct the final cDNA libraries for paired-end sequencing using the Illumina HiSeq 2500 system (Biomarker Technologies Co., Ltd, Beijing, China).

Raw reads from each sample were processed by remove primer and adaptor sequences. Low quality reads were filtered out and the clean reads consist of more than 80% base-pair with Q-value≥30. The clean reads were aligned with TopHat software [27] using the *Brassica* Database (http://brassicadb.org/brad/) as the reference and were assembled into contigs. By performing pair-end joining and gap filling, contigs were assembled and clustered to obtain unique reads. The saturation of reads in each library was evaluated by comparing the number of identified genes versus total reads.

### Analysis of gene expression and functional annotation

Gene expression in each sample was estimated by calculating read density as ‘reads per kilobase per million mapped reads’ (RPKM) [28]. Differentially expressed genes (DEGs) between different samples were identified using EBSeq software [29]. A threshold for false discovery rate (FDR) < 0.01 and fold change (FC) ≥ 2 were used to determine significant differences in gene expression. Genes with FC < 2 were not considered to be DEGs and were therefore discarded.

DEG sequences were compared with the non-redundant protein (Nr, NCBI), Swiss-Prot, Gene Ontology (GO, http://www.geneontology.org/), Clusters of Orthologous Groups (COG) and Kyoto Encyclopedia of Genes and Genomes (KEGG) databases using BLAST software, and information about the DEGs was annotated. GO and KEGG pathway analyses were performed based on the GO and KEGG databases. DEGs at both stages (in cold treatment ending and before flower bud differentiation) were investigated in detail.

### Quantitative Real-time PCR

To validate the RNA-Seq results, Quantitative Real-time PCR (qPCR) analysis was performed use gene-specific primers for two randomly selected genes. For reverse transcription, the concentration of each RNA sample was adjusted to 500 ng/μL. Single-stranded cDNA was synthesized using a PrimeScript^®^ RT reagent kit (Perfect Real Time; TaKaRa, DRR037A) following the manufacturer’s instructions. Specific primers used for qPCR were designed using Primer 3 software according to gene sequence, and the pak choi *ACTIN* gene was chosen as an internal reference gene. qPCR was carried out using SYBR^®^ *Premix Ex Taq* ^™^ II (Tli RNaseH Plus; TaKaRa, RR820A) in a 25-μl reaction volume. The reaction mixture included 20 ng cDNA (2 μL), 8.5 μL ddH_2_O, 12.5 μL SYBR and 2 μL primers. The program was as follows: 95°C for 3 min, 40 cycles of 95°C for 15 s, 55°C for 30 s and 72°C for 30 s. The relative expression levels were calculated using the ΔΔCt method [30].

### Data access

The transcriptome sequencing data from this study have been deposited in the NCBI SRA database and are accessible through accession numbers SRP064332 (http://www.ncbi.nlm.nih.gov/sra).

## Results

### Analysis of read saturation in each library

**Fig 1:**
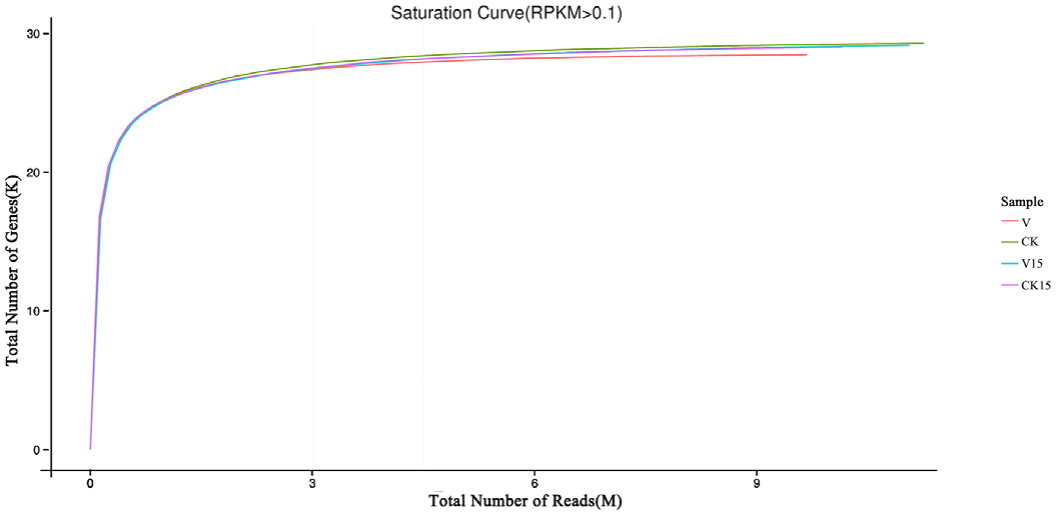
The saturation of reads in each library.

The saturation of reads in each library was evaluated by comparing the number of identified genes with the total number of reads. The results are shown in Fig 1. The number of new genes detected decreased with increasing number of reads in all four datasets. No new genes were found when the number of reads approached 8M, and each dataset produced 9M reads. These results suggested that all genes had already been analyzed during sequencing, and the sequences reached saturation, thereby providing enough information for subsequent DEG analysis.

### Quality assessment of the sequencing results

The results of statistical analysis of raw data for each sample after sequencing are shown in Table 1. A total of 11,111,314, 11,268,910, 11,072,438 and 10,169,936 reads were sequenced from the V, CK, V15 and CK15 libraries, respectively. Clean reads obtained by filtering out adaptor sequences, contaminating sequences and low quality reads accounted for 87.09%, 99.86%, 99.90% and 99.86% of total reads in the four libraries, respectively, which indicates that the quality of sequencing was excellent. Single-copy tags in each library were excluded from further analysis. Finally, a total of 5,120,186, 5,672,735, 4,792,362 and 4,350,258 clean reads were clustered into 924,027 (V), 1,152,144 (CK), 1,317,199 (V15) and 1,190,408 (CK15) unique reads, respectively.

**Table 1:** Reads of each dataset and mapping results.

Read type	In cold treatment ending		Before flower bud differentiation	
	V	CK	V15	CK15
Total reads	11,111,314	11,268,910	11,072,438	10,169,936
Clean reads	9,676,674 (87.09%)	11,253,403 (99.86%)	11,061,770 (99.90%)	10,156,073 (99.86%)
Clean read copy 1	4,546,483	5,580,668	6,269,408	5,805,815
Unique reads	924,027	1,152,144	1,317,199	1,190,408
Mapped reads	8,025,626	9,543,109	9,411,660	8,609,294
Unique mapped reads	6,915,600	8,741,375	8,827,419	8,009,239
Multiple mapped reads	1,110,026	801,734	584,241	600,055

The clean reads from each library were aligned to the Chinese cabbage genome. A total of 82.94%, 84.80%, 85.08% and 84.77% of clean reads were matched to the reference genome for V, CK, V15 and CK15, respectively, including unique mapped reads and multiple mapped reads. The proportion of mapped genes was high, indicating that the sequences and reference genome are suitable for further analysis. The unique mapped reads accounted for 86.17%, 91.60%, 93.79% and 93.03% of total mapped reads in each library and could therefore been used for further DEG analysis.

### Identification of differentially expressed genes during vernalization

Unique reads that perfectly matched reference genes in each library were normalized to RPKM and used to evaluate the expression levels. Genes with less than two-fold differences in expression between the V and CK libraries at both stages were excluded from further analysis.

The DEGs analysis was conducted for the unique reads which were mapped to the reference genome between low temperature and control treatment libraries at two stages (in cold treatment ending and before flower bud differentiation). The number of genes with different fold changes in expression was shown in Table 2. The number of DEGs in cold treatment ending was 1,664, including 747 up-regulated and 917 down-regulated genes, while before flower bud differentiation, there were 1,192 DEGs, including 533 up-regulated and 659 down-regulated genes. Comparing DEGs between the two stages, 42 genes exhibited similar expression patterns in the two treatments; these genes were down-regulated and are mainly involved in meristem initiation, glycolysis and gluconeogenesis.

**Table 2:** Differentially expressed genes in two stages during vernalization.

		In cold treatment ending	Before flower bud differentiation	Common to both
		CK vs V	CK15 vs V15	
DEG (>2 fold)	Total	1,664	1,192	42
	Up	747	533	17
	Down	917	659	25
DEG (2–10 fold)	Total	1,395	1,111	40
	Up	675	486	16
	Down	720	625	24
DEG (10–50 fold)	Total	251	79	2
	Up	68	46	1
	Down	183	33	1
DEG (>50 fold)	Total	18	2	0
	Up	4	1	0
	Down	14	1	0

### Functional annotation of DEGs after vernalization

The GO database can be applied to any species to identify the functions of genes and protein. Based on the overall analysis of gene expression profiles presented above, DEGs between low temperature and control treatment in cold treatment ending (after cold treatment) and before flower bud differentiation periods were annotated using GO functional classification analysis. More than 90% of DEGs at each stage were annotated. The proteins encoded by the DEGs are mainly located in the extracellular region, cell junction and extracellular matrix. These proteins exhibit electron carrier activity, antioxidant activity, binding protein/transcription factor activity and nutrient reservoir activity, and they participate in signal conduction, immune system processes, development and death and rhythmic processes.

**Fig 2:**
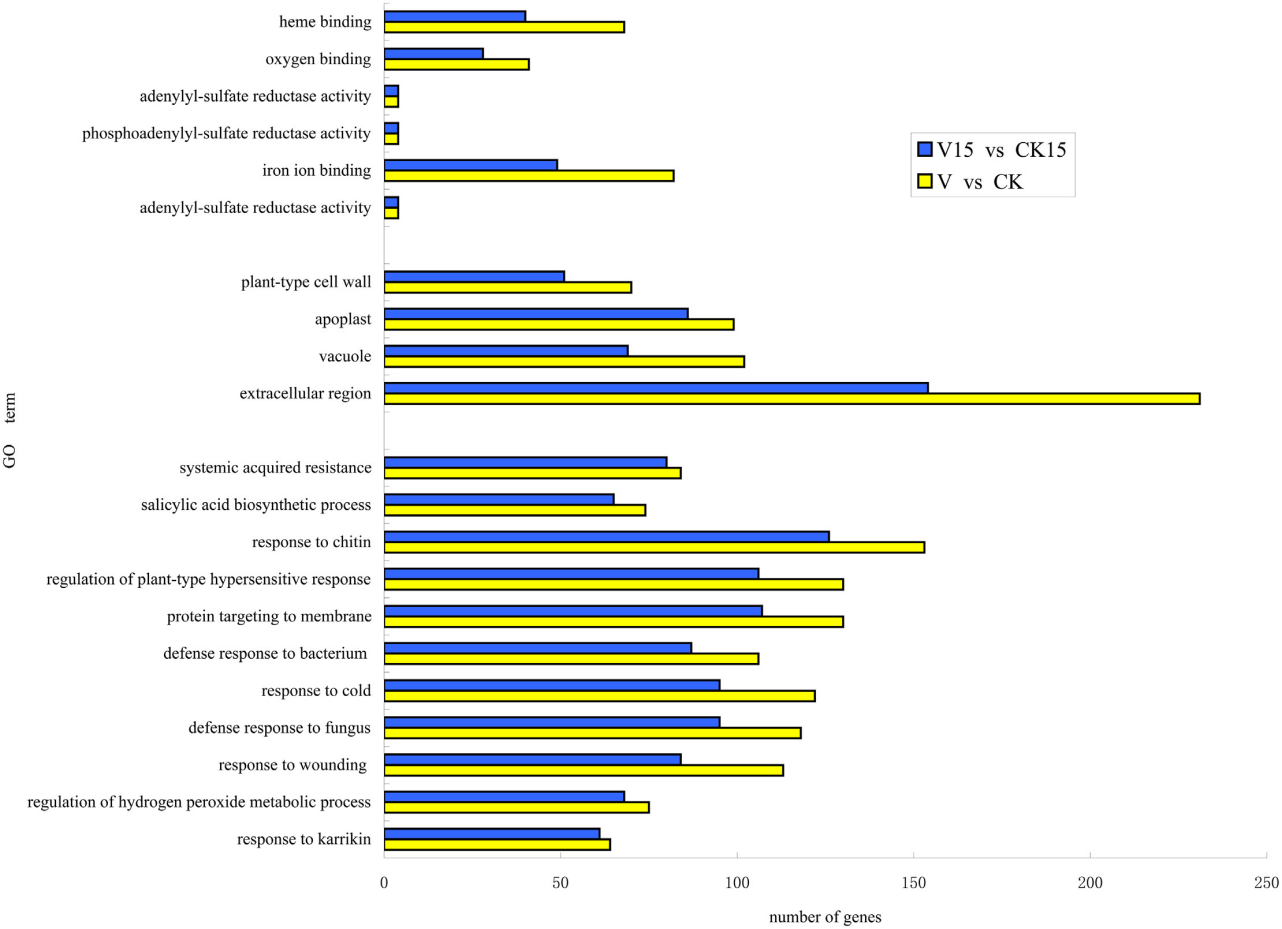
GO enrichment analysis of DEGs after vernalization. A threshold of corrected p-value≤0.05 was used to judge the significantly enriched GO terms in DEGs. V15 vs CK15: DEGs between low temperature treatment and control samples before flower bud differentiation; V vs CK: DEGs between low temperature treatment and control samples in cold treatment ending.

The functions of DEG filtered by p-value < 0.05 in cold treatment ending and before flower bud differentiation were classified (Fig 2) by GO analysis. The numbers of the DEGs, whose encoded protein located in extracellular region in each stage, were 231 and 154, accounting for 13.76% and 12.90% of all DEGs, respectively. Functions of these genes were analyzed and found that 203 and 129 genes are involved in the categories iron ion binding, oxygen binding and heme binding, accounting for 11.38% and 9.80% of all DEGs in the first and second stage, respectively. They mainly participated in biological processes including the protein targeting to membranes, regulation of plant-type hypersensitive response and respond to chitin.

To further elucidate the specific functions of the DEGs, genes with more than 50-fold changes in expression were analyzed. There were 18 DEGs in cold treatment ending, including four up-regulated genes and 14 down-regulated genes. Among these, Bra014527 was up-regulated after vernalization. The homologous gene in *Nicotiana tabacum* encodes the mitogen-activated protein kinase Ntf4-2, while in *A*. *thaliana* the gene is AT3G59790, it encodes a member of the MAP kinase family and is transiently expressed at leaf tips, loss of this gene results in late flowering in long-day conditions [31]. Other highly expressed genes and their functions are shown in Table 3. Two DEGs exhibited more than 50-fold changes in expression before flower bud differentiation between loe temperature-treated and control samples, including one upregulated gene and one downregulated gene. Among these Bra013113 was up-regulated after cold treatment, its homologue in *A*. *thaliana* encodes a hypothetical protein that participates in inositol hexaphosphoric acid biosynthesis, while homologous gene of down-regulated Bra018216 in *A*. *thaliana* encodes a heat shock protein and is induced in response to heat and high light intensity.

**Table 3:** DEGs with more than 50-fold changes in expression at both stages of vernalization.

Stage	Gene expression	Gene name	Log2 FC	Gene annotation
In cold treatment ending	Up	Bra014527	6.95	MAP kinase family
		Bra034235	5.99	CYP71B4
		Bra000194	5.89	alpha-expansion protein
		Bra036724	5.80	hypothetical protein
	Down	Bra011746	- 8.92	cysteine proteinase1
		Bra010592	- 7.95	cysteine proteinase1
		Bra031485	- 6.46	hypothetical protein
		Bra004430	- 6.32	tryptophan-rich sensory protein-like protein
		Bra030917	- 6.23	lipase SIL1
		Bra003004	- 6.07	protochlorophyllide reductase A
		Bra003699	- 6.05	extensin—rape
		Bra008720	- 5.97	uncharacterized protein
		Bra004429	- 5.94	rubber elongation factor protein
		Bra031301	- 5.91	isocitrate lyase
		Bra036259	- 5.88	glutathione S-transferase 3
		Bra039006	- 5.78	putative retroelement pol polyprotein
		Bra010393	- 5.68	predicted protein
		Bra009105	- 5.68	peroxidase
Before flower bud differentiation	Up	Bra013113	6.42	hypothetical protein
	Down	Bra018216	-5.64	heat shock protein

Using genes related to vernalization in *A*. *thaliana* as the reference, we analyzed the differences in expression of homologous genes from pak choi between low temperature treatment and control conditions at both stages. In *A*. *thaliana*, AT4G30200 encodes a protein with similarity to VRN5 and VIN3 and promotes flowering by silencing *FLC*. The homologous gene Bra024097 in pak choi was up-regulated before flower bud differentiation in low temperature treatment sample. AT5G61150 in *A*. *thaliana* encodes highly hydrophilic protein involved in positively regulating *FLC* expression, while its homologous gene Bra035940 in pak choi was down-regulated in low temperature-treated samples at both stages. These results suggested that these two genes may participate in the process of vernalization in pak choi, and they should therefore be subjected to further analysis.

### Metabolic pathway analysis of DEGs in response to vernalization

The functions of different genes are coordinated to allow them to carry out biological functions in an organism. Pathway analysis of the DEGs was conducted to further elucidate their functions. The results of KEGG pathway enrichment analysis of DEGs (at p-value < 0.5) are shown in Table 4. The DEGs in cold treatment ending and before flower bud differentiation were enriched in the pathways linoleic acid metabolism and tryptophan metabolism. The DEGs in cold treatment ending were also enriched in the pathways of nitrogen metabolism and phenylalanine metabolism, while the DEGs before flower bud differentiation were enriched in the pathways of carotenoid biosynthesis and glucosinolate biosynthesis.

**Table 4:** KEGG enrichment analysis of DEGs.

Stage	Pathway	ko_ID	P-value
In cold treatment ending	Phenylpropanoid biosynthesis	ko00940	3.89E-07
	Phenylalanine metabolism	ko00360	5.86E-06
	Nitrogen metabolism	ko00910	1.87E-06
	Linoleic acid metabolism	ko00591	0.00085
	alpha-Linolenic acid metabolism	ko00592	0.00454
	Stilbenoid, diarylheptanoid and gingerol biosynthesis	ko00945	0.00624
	Tryptophan metabolism	ko00380	0.02471
Before flower bud differentiation	Glucosinolate biosynthesis	ko00966	0.00007
	Carotenoid biosynthesis	ko00906	0.00583
	Tryptophan metabolism	ko00380	0.00817
	Linoleic acid metabolism	ko00591	0.022504

All metabolic pathways in which the DEGs are involved were analyzed, revealing that many DEGs participate in hormone metabolism and carbohydrate metabolism. After vernalization, the genes related to the biosynthesis of gibberellin (GA) such as Bra016763, Bra028186, Bra009285 were largely up-regulated, and the genes involved in the biosynthesis of salicylic acid (SA) (Bra025728), response to SA (Bra017839) and SA-mediated signaling pathway (Bra021110) were all up-regulated. Many genes related to the sucrose response and sucrose-mediated signaling pathway such as Bra021110, Bra011656 and Bra001600 were up-regulated as well.

### Validation of DEGs by quantitative real-time PCR

**Fig 3:**
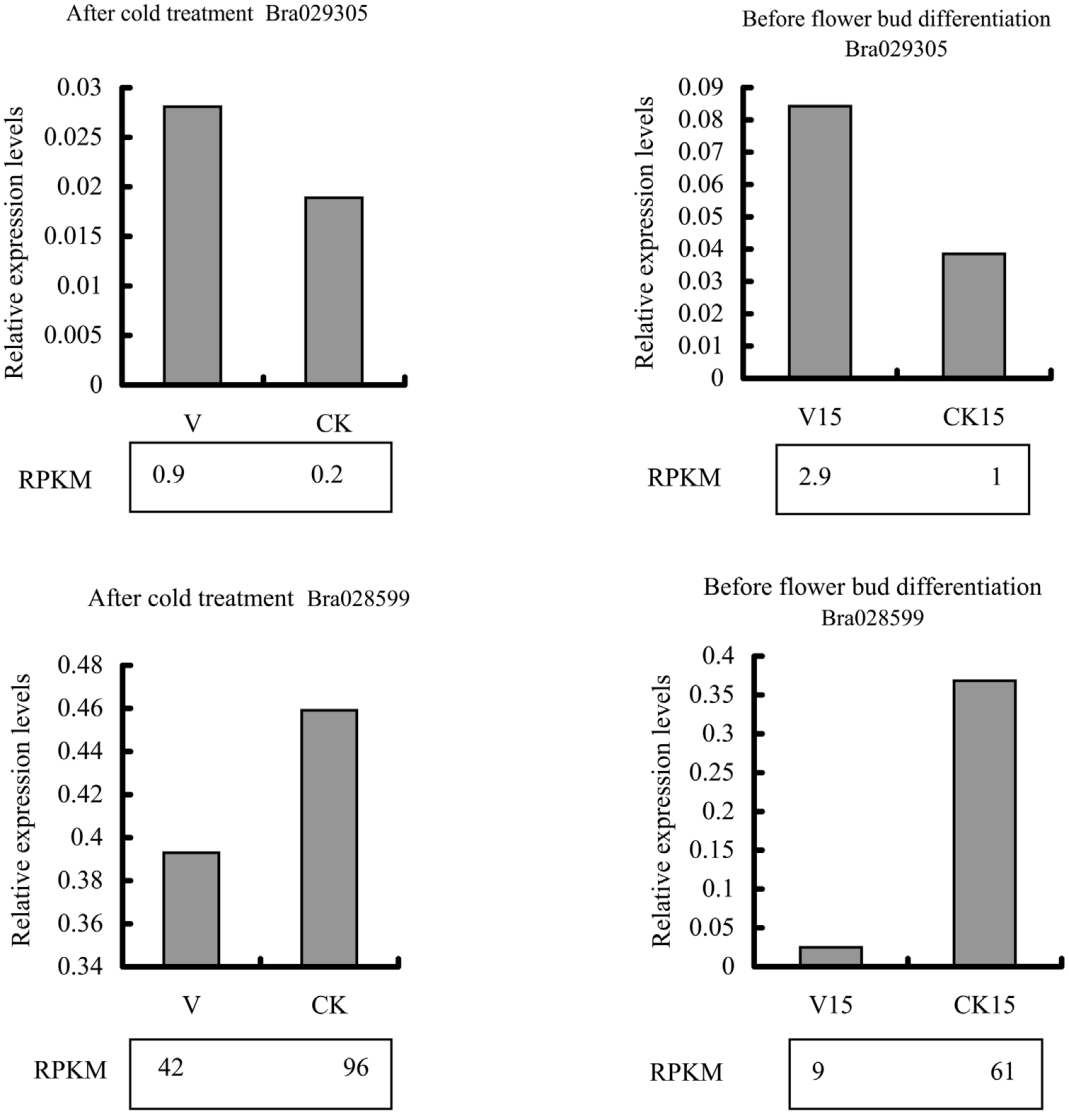
RT-qPCR validation of RNA-Seq results based on gene expression levels. RPKM: reads per kb per million reads.

To validate the RNA-Seq data, gene Bra028599 and Bra029305 were selected for qPCR analysis at both stages of vernalization; the results are shown in Fig 3. The expression patterns of these genes obtained by qPCR and RNA-Seq are similar, indicating that the results from RNA-Seq are believable.

## Discussion

Under low temperature conditions, different metabolic and signal transduction pathways are regulated in the plant through altered gene expression. Thus, genes function in the response to vernalization at the transcriptional and translational levels and the plant eventually flowers during the appropriate period. In this study, informations about the expression of 41,020 genes in pak choi in response to vernalization were obtained using RNA-Seq. 1664 and 1192 DEGs were screened out in cold treatment ending and before flower bud differentiation respectively by comparing the copy of the genes detected between cold treatment and control. Compared to the control, after vernalization, newly expressed or silenced genes accounted for 5.27% and 1.72% of the total genes detected at the two stages examined, respectively. Most genes showed altered expression levels after vernalization.

Vernalization is a process in which flowering is promoted by prolonged low temperature treatment. The meristem of the shoot apex has the potential to produce flowers after vernalization, and the plant will bloom when the temperatures warm [32]. Genes such as *FRI*, *FLC*, *VRN1*, *VRN2* and *VIN3* are involved in the vernalization process in *Arabidopsis thaliana* [33]. FLC is an inhibitor of the blooming process, and the presence of a dominant allele of *FRI* enhances the inhibitory effect of FLC [34, 35]. *VIN* participates in the suppression of *FLC* expression at the beginning of the vernalization process, and *VRN1* and *VRN2* help maintain the vernalization state [36]. The meristems of the shoot and root apex perceive low temperature conditions and the crucial role of vernalization is suppressing the expression of *FLC*. In our obtained data, the homologous genes of *FLC* from Pakchoi, Bra006051 and Bra009055, were down-regulated after vernalization, while Bra020445, a homologue of *VIN3*, and Bra001729, a homologue of *VRN1*, were up-regulated after vernalization. These results are consistent with the results of previous studies, which help confirm the accuracy of our data.

The expression patterns of 42 genes examined were similar at both stages. These genes primarily participate in hormone metabolism, glucide metabolism, meristem initiation and transition of the meristem from the vegetative to reproductive stage. GA promotes flowering in Chinese cabbage [37], pak choi [38] and radish [39]. In this study, genes related to GA biosynthetic and catabolic processes in pak choi were upregulated at both stages after vernalization, indicating that GA metabolism is active under vernalization, which is in agreement with previous findings[37]. In addition, many genes responsive to SA and involved in SA-mediated signal transduction pathways were upregulated in response to low temperature, indicating that SA biosynthesis is enhanced under vernalization. SA plays a vital role in plant resistance to disease [40], salinity and low temperature stress [41] and especially drought resistance [42, 43]. However, the role of SA in vernalization remains unclear, but it may be related to cold stress. Many genes that function in glucide metabolism, which play a role in the response to sucrose and are related to the sucrose-mediated signal transduction pathway, were upregulated under cold treatment, indicating that sucrose-related pathways are promoted under vernalization. Some genes involved in glucide metabolism were upregulated under low temperature treatment, but some were downregulated, indicating that the proportion of different sugars is altered during vernalization. Genes that function in regulating meristem growth and meristem initiation were downregulated during vernalization, perhaps because plant growth slows under low temperature conditions.

Glucosinolates are nitrogen- and sulfur-containing secondary metabolic substances (primarily found in the *Brassicaceae* family) that plays an important role in plant defense respond to insects and pathogens and in plant chemical allelopathy [44, 45]. Glucosinolates can be divided into three classes based on the similarity of their amino acid precursors: methionine, phenylalanine and tryptophan [46]. In *Arabidopsis thaliana*, glucosinolates were distributed in reproductive organs (inflorescence, silique, seed), as well as vegetative organs (root, stem, leaf). Dormant and germinating seeds have the highest concentrations of these substances, followed by inflorescences, siliques (fruits), leaves and roots [47]. In our study, genes related to glucosinolate biosynthesis were downregulated after low temperature treatment at both stages, indicating that the accumulation of glucosinolate in pak choi is reduced in response to vernalization. These results suggested that glucosinolates may function during vernalization in pak choi, but their specific function remains to be analyzed.

## Conclusion

Using RNA-Seq technology, the gene expression profiles of the shoot apex after cold treatment were analyzed in low temperature and control treatment pakchoi. A total of 1,664 and 1,192 differentially expressed genes in pak choi were detected between samples in cold treatment ending and before flower bud differentiation periods, respectively, and among which 42 genes had similar expression trend at both stages. Detailed annotation revealed that the proteins encoded by the DEGs are mainly distributed in the extracellular region, cell junction and extracellular matrix. They possess electron carrier activity, antioxidant activity and binding protein/transcription factor activity and are involved in signal transduction and the immune system process. The DEGs were enriched in the categories phenylalanine metabolism, tryptophan metabolism and glucosinolate biosynthesis. The homologues to vernalization-related genes in *Arabidopsis thaliana* were analyzed in pak choi and three vernalization-related pak choi genes, Bra014527, Bra024097 and Bra035940 were discovered. These results will facilitate further research on the vernalization mechanism in pak choi.
